# Transition-Metal-Free Highly Efficient Aerobic Oxidation of Sulfides to Sulfoxides under Mild Conditions

**DOI:** 10.3390/molecules15010083

**Published:** 2009-12-28

**Authors:** Hua Zhang, Chunyu Chen, Renhua Liu, Qiang Xu, Weiqie Zhao

**Affiliations:** 1 State Key Laboratory of Fine Chemicals, School of Chemical Engineering, Dalian University of Technology, No.158-139, Zhongshan Road, Dalian 116012, China; E-Mails: chenchunyu321@yahoo.cn (C.Y.C.); xuqianguser@163.com (Q.X.).; zyzhao@chem.dlut.edu.cn (W.Q.Z.); 2 School of Pharmacy, East China University of Science and Technology, Shanghai 200237, China; E-Mail: liurh@ecust.edu.cn (R.L.)

**Keywords:** aerobic oxidation, sulfids, Br_2_, NaNO_2_, molecular oxygen

## Abstract

A highly efficient transition-metal-free catalytic system Br_2_/NaNO_2_/H_2_O has been developed for a robust and economic acid-free aerobic oxidation of sulfides. It is noteworthy that the sulfide function reacts under mild conditions without over-oxidation to sulfone. The role of NaNO_2_ as an efficient NO equivalent for the activation of molecular oxygen was identified. Under the optimal conditions, a broad range of sulfide substrates were converted into their corresponding sulfoxides in high yields by molecular oxygen. The present catalytic system utilizes cheap and readily available agents as the catalysts, exhibits high selectivity for sulfoxide products and releases only innocuous water as the by-products.

## 1. Introduction

The oxidation of sulfides into the corresponding sulfoxides is one of the most important functional group transformations in organic synthesis [1]. Sulfoxides as a class of important compounds are generally used as synthetic intermediates for the construction of various chemically and biologically significant molecules [2,3], as well as for the synthesis of drugs and natural products [3,4,5], Therefore, many efficient methods have been developed for the oxidation of sulfides to sulfoxides. However, it is difficult to control the results while the sulfoxides could be further oxidized to sulfones. A large number of methods have been developed to overcome this drawback; however, most of these reactions require a stoichiometric amount of oxidant, resulting in undesirable waste [6,7,8]. Hydrogen peroxide and molecular oxygen has recently been utilized as an attractive and environmentally benign oxidant for the oxidation of sulfides, because they are inexpensive, easy to handle, safely stored, and produce only water as a side-product [9,10,11,12]. Hydrogen peroxide is usually applied in catalytic sulfide oxidations system since it is an environmentally benign oxidant [13,14,15,16,17,18]. However, selective oxidation by molecular oxygen is even more attractive because of its obvious economical and environmental advantages [19,20,21,22].

**Scheme 1:**
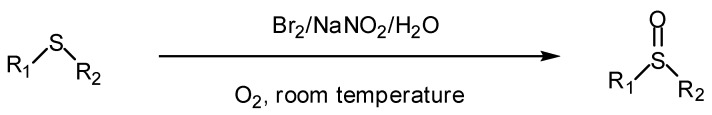
Oxidation of sulfides to the corresponding sulfoxides.

Recently, the use of molecular oxygen as terminal oxidant has received great attention for both economic and environmental benefits, and many highly efficient systems have been developed for catalytic aerobic sulfide oxidation using rhenium [23], palladium [24], ruthenium [25], copper [26,27] manganese [26], iron [27,28,29], cobalt [30] and gold-based catalysts [31]. However, the metal catalysts can be expensive and may hinder the purification of products, and cause some environment problems. Only a few catalyst systems, for example, small amounts of cheap metal salts, provide an efficient catalyst for aerobic oxidation of sulfides under mild conditions. Therefore, the development of an efficient nonmetallic catalyst for the aerobic oxidation of sulfides under mild conditions has long been desired. Our research was inspired by the mechanism of selective catalysis of thioether oxidations with dioxygen by Bosch *et al*. [32] who reported the facile and selective oxidation of various aliphatic and aromatic sulfides by the combination of NO_2_ and dioxygen at room temperature and below. The catalysis of thioether autoxidation was also effected by small amounts of other nitrogen oxides, such as nitric oxide and nitrosonium salts. NaNO_2_ as an alternative NO source for their property were identified as an efficient NO equivalent for the activation of molecular oxygen [33,34]. In this context, a process for the highly efficient aerobic oxidation of a wide range of sulfides utilizing a DBDMH/NaNO_2 _and HBr/t-BuONO catalyst system that was free from any transition-metal co-catalysts has been disclosed by our research group [35,36]. On the other hand, the halogens and their derivatives are considered environmentally unfriendly, despite their advantages such as low price, easy handling, commercial availability, and relatively high stability. The biggest disadvantage of the utilization of molecular bromine in the oxidation of sulfides is the formation of byproducts such as sulfonic or sulfinic acids and bromosubstituted sulfides or sulfones [37]. If Br_2_ was only used in catalytic amounts but not in stoichiometric amount, the transformation of sulfides into the corresponding sulfoxides could be more attractive and effective oxidation procedure using molecular oxygen. A novel metal-free catalytic system Br_2_/NaNO_2_/H_2_O for the highly selective oxidation of a variety of sulfides using molecular oxygen as the terminal oxidant in a small amount of water at ambient temperature (Scheme 1) is reported in this paper.

## 2. Results and Discussion

Initial investigation of aerobic oxidation was carried out using methyl phenyl sulfide as a substrate with 3 mol % of Br_2_ and oxygen under 25 °C for 1 h. The preliminary result (21% conversion) clearly indicated the role of Br_2_ as an active catalyst. We sought to find a co-catalyst to bridge the gap between O_2_ activation and HBr reoxidation.The ready availability and unique redox property of NaNO_2_ as a source of NO under acidic conditions attracted our attention. Although NaNO_2 _alone showed little activity (10% conversion), when 3 mol % of Br_2_ and 5 mol % of NaNO_2 _were both employed in aerobic oxidation, a highly efficient catalyst system emerged. The keys to the newly developed oxidation process are the discovery of molecular oxygen being able to oxidize HBr to Br_2_ *in situ* with NaNO_2_ as the catalyst and its suitability in an aqueous environment (Table 1).

**Table 1:** The catalysts effect on the reaction.^ a^

Entry	NaNO_2_ (mol %)^b^	Br_2_ (mol %)^ b^	Conversion (%) ^c^
1	0	3	21
2	5	0	10
3	5	3	100

^a ^Reaction conditions: methyl phenyl sulfide (MPS) (10 mmol), O_2_ (80 mL/min), 25 °C, H_2_O(1.5 mL); ^b^ Br_2_ (0.3 mmol, 0.015 mL), NaNO_2_ (0.5 mmol,0.0345 g); ^c^ Conversions were determined by GC and counted with area normalization.

### 2.1. Aerobic oxidation of sulfides to sulfoxides

Ten mmol substrates of analogues of methyl aryl sulfides were reacted with 5 mol % of NaNO _2_ in the presence of 3–5 mol % Br_2_, 1.5 mL of water and oxygen (80 mL/min) in CH_3_CN under 25 °C in 1 h. The reactions were took place efficiently (Table 2).

**Table 2:** Catalytic aerobic oxidation of sulfide in a small amount of water.^ a^

Entry	Substrate	Product	Time (h)	Br_2_ (mol %)	Conversion (%)^b^	Yield (%)^c^
1	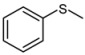	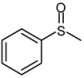	1	3	100	96
2	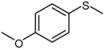	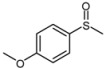	1	3	100	92
3	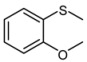	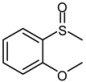	1	3	100	95
4	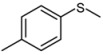	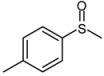	1	3	100	94	
5	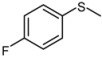	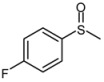	1	3	100	95	
6	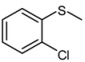	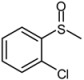	1	3	100	94	
7	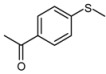	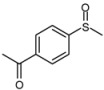	1	3	100	93	
8	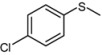	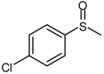	2	3	100	92	
9	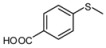	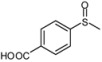	5	5	100	96	
10	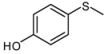	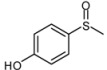	5	5	25	21^d^	
11	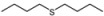	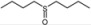	1	3	100	92	

^a ^Reaction conditions: substrates (10 mmol), NaNO_2_ (0.5 mmol,0.0345 g), H_2_O (1.5 mL), CH_3_CN (20 mL), O_2_ (80 mL/min), 25 °C; ^b ^Conversions were determined by GC with area normalization; ^c^ isolated yields; **^ d^** yield were determined by GC with area normalization.

**Scheme 2:**
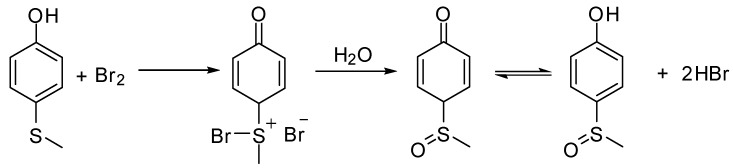
The catalytic oxidation of 4-(methylthio)phenol.

As shown in Table 2, most of methyl aryl sulfides including electron-rich and nucleophilic-rich aromatic compounds were converted into their corresponding sulfoxides in high isolated yields within 1 h (entries 1–7). 4-Chlorophenyl methyl sulfide and 4-(methylthio)benzoic acid showed relatively low activities in the oxidation. To address this, we prolonged the reaction time to 2 h, 4-Chlorophenyl methyl sulfide was thus converted into 4-chlorophenyl methyl sulfoxide with 100% conversion in high isolated yields (entry 8). Increasing the amount of Br_2_ (to 5 mol %) in the catalyst system will improve the reaction rate－the oxidation of 4-(methylthio)benzoic acid could be completed within 5 h (entry 9). Simple alkyl sulfides such as dibutyl sulfide were also efficiently oxidized (entry 11). It is noteworthy that function selective oxidation can be achieved with this method, which is generally problematic in classical oxidation processes. Under the modified conditions (Br_2_, 5 mol %), the conversion of 4-(methylthio)phenol (entry 10) is only about 25% within 5 h. Recognizing the oxidation of 4-(methylthio)phenol by molecular oxygen to the corresponding 4-(methylsulfoxyl)phenol, an intermediate may be via conjugate transformation converted into a similar-benzoquinone compound, then the ultimate compound is obtained by conjugate match once more. The key of the reaction is the form of the intermediate, the more difficult the intermediate formed, the lower conversion the product received (Scheme 2) [38].

### 2.2. Mechanism of aerobic sulfide oxidation

**Scheme 3:**
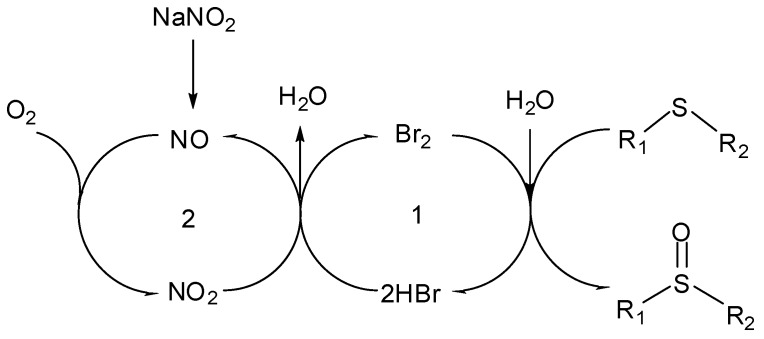
Overall Catalytic Mechanism.

A possible overall mechanism of this new and transition-metal-free catalytic oxidation can be described by the dual cycle in Scheme 3. Thus, transformation of sulfides into their corresponding sulfoxides is fulfilled by the coupling of multiple redox reactions. Initially the electrophilic attack of Br_2_ on sulfur in the organic phase leads to the sulfoxide [38]. The running of Cycle 1 continuously produces the reoxidation of HBr to Br_2_ by NO_2 _[39], which is the key for a catalytic amount of Br_2_ to benefit the oxidations of active sulfide substrates. NO_2_ is reduced to NO when it completes the oxidation of HBr. NO is easily released from NaNO_2_ as an efficient NO equivalent .The oxidation of NO into NO_2_ is a process easily accomplished with molecular oxygen (Cycle 2), and the coupling of the two cycles furnishes a novel, coherent, and efficient aerobic sulfide oxidation system.

## 3. Experimental

### 3.1. Materials

All methyl aryl sulfides (Wuling Science and Technology&Industry Co. Ltd.) and their analogues (Zhejiang Shou & Fu Chemical Co. Ltd), dibutyl sulfide (Shandong Teng & Wu Spice Co.Ltd), was a commercial product used as received without any further purification. Deionised water was used in all experiments. All other solvents used were analytical grade.

### 3.2. Instrumentation

IR spectra were recorded on a Nicolet 20DXB FT-IR instrument. ^1^H-NMR spectra were recorded on a Varian Inova 400MHz spectrometer. HPLC chromatograms were recorded with an Agilent 1100 system. GC chromatogram was recorded on a Varian 3700 apparatus. MS spectra was recorded on a HP 6890/5973 instrument. 

### 3.3. General procedure for catalytic aerobic sulfides oxidation

Oxidation of methyl phenyl sulfide is described as a representative example of aromatic compounds: in a 100 mL Schlenk flask equipped with a mechanical stirrer were placed methyl phenyl sulfide (10 mmol), acetonitrile (20 mL), water (1.5 mL), Br_2 _(3 mol %) and sodium nitrite (5 mol %). The reaction mixture was stirred at 25 °C under a continuous stream of oxygen (80 mL/min). Upon completion after 1 hour, as detected by GC, the solvent was removed and the residue extracted with ethyl acetate. The organic extract was first washed with 5% sodium bicarbonate solution, then the organic layer was finally dried over anhydrous sodium sulfate. The yield was calculated on the basis of 10 mmol of substrate. The ^1^H-NMR spectrum was recorded directly using the isolated product.

### 3.4. Spectroscopic data for products

*Methyl phenyl sulfoxide* (Table 2, entry 1): Yellow liquid; ^1^H-NMR (DMSO-d6): *δ* 2.73 (3H, s, CH_3_), 7.52-7.59 (3H, m, Ph), 7.67-7.71(2H, m, Ph); IR (KBr, cm^-1^): ν 1048, 749, 692; MS (EI), *m/z*: 140 [M]^+^.

*4-Methoxyphenyl methyl sulfoxide* (Table 2, entry 2): Yellow solid; ^1^H-NMR (DMSO-d6): *δ* 2.71(3H, s, SOCH_3_), 3.83 (3H, s, OCH_3_), 7.13 (2H, d, *J* = 8.8 Hz, Ph), 7.63 (2H, d, *J* = 8.8 Hz, Ph); IR (KBr, cm^-1^): *ν* 1027, 831; MS (EI), *m/z*: 170 [M]^+^.

*2-Methoxyphenyl methyl sulfoxide* (Table 2, entry 3): Yellow liquid; ^1^H-NMR (DMSO-d6): *δ* 2.70 (3H, s, SOCH_3_), 3.86 (3H, s, OCH_3_), 7.14 (1H, d, *J* = 7.6 Hz, Ph), 7.21 (1H, t, *J* = 7.6 Hz, *J* = 7.4 Hz, Ph), 7.52 (1H, t, *J* = 7.4 Hz, *J* = 8.3 Hz, Ph), 7.63(1H, d, *J* = 8.3 Hz, Ph); IR (KBr, cm^-1^): *ν* 1038, 757; MS (EI), *m/z*: 170 [M]^+^.

*4-Methylphenyl methyl sulfoxide* (Table 2, entry 4): Yellow solid; ^1^H-NMR (DMSO-d6): *δ* 2.35 ((3H, s, CH_3_), 2.69 (3H, s, SOCH_3_), 7.4 (2H, d, *J* = 8.4 Hz, Ph), 7.56 (2H, d, *J* = 8.4 Hz, Ph); IR (KBr, cm^-1^): *ν* 1046, 810; MS (EI), *m/z*: 154 [M]^+^.

*4-Fluorophenyl methyl sulfoxide* (Table 2, entry 5): Yellow solid; ^1^H-NMR (DMSO-d6): *δ* 2.73 (3H, s, CH_3_), 7.40 (2H, t, *J* = 9.0 Hz, *J* = 8.8 Hz, Ph), 7.74 (2H, q, *J* = 8.8 Hz, *J* = 5.4 Hz, Ph); IR (KBr, cm^-1^): *ν* 1048 , 834; MS (EI), *m/z*: 158 [M]^+^.

*2-Chlorophenyl methyl sulfoxide* (Table 2, entry 6): Yellow liquid;^ 1^H-NMR (DMSO-d6): *δ* 2.81 (3H, s, CH_3_), 7.56-7.59 (m, 2H), 7.62~7.68 (m, 1H), 7.81-7.86 (m, 1H); IR (KBr, cm^-1^): *ν* 1067, 759; MS (EI), *m/z*: 174 [M]^+^.

*1-(4-Methansulfinylphenyl)-ethanone* (Table 2, entry 7): Yellow solid; ^1^H-NMR (DMSO-d6): *δ* 2.63 (3H, s, COCH_3_), 2.80 (3H, s, SOCH_3_), 7.83 (2H, d, *J* = 8.4 Hz, Ph), 8.13 (2H, d, *J* = 8.4 Hz, Ph); IR (KBr, cm^-1^): *ν* 1674, 1047, 830; MS (EI), *m/z*: 182 [M]^+^.

*4-Chlorophenyl methyl sulfoxide* (Table 2, entry 8): Yellow solid; ^1^H-NMR (DMSO-d6): *δ* 2.78 (3H, s, CH_3_), 7.67 (2H, d, *J* = 8.4 Hz, Ph), 7.73 (2H, d, *J* = 8.4 Hz, Ph); IR (KBr, cm^-1^): *ν* 1051, 822; MS (EI), *m/z*: 174 [M]^+^.

*4-Methanesulfinylbenzoic acid* (Table 2, entry 9): White powder; ^1^H-NMR (DMSO-d6): *δ* 2.79 (3H, s, CH_3_), 7.81 (2H, d, *J* = 8.0 Hz, Ph), 8.11 (2H, d, *J* = 8.0 Hz, Ph), 13.30 (1H, s, COOH); IR (KBr, cm^-1^): *ν* 2773, 1690, 1083, 856; MS (EI), *m/z*: 184 [M]^+^.

*Dibutyl sulfoxide* (Table 2, entry 11): Yellow liquid; ^1^H-NMR (DMSO-d6): *δ* 0.91 (3H, t, CH_3_), 1.41 (2H, m, CH_2_), 1.608 (2H, m, CH_2_), 2.657 (2H, m, SOCH_2_); IR (KBr, cm^-1^): *ν* 737, 923, 1021, 1085; MS (EI), *m z*: 162 [M]^+^.

## 4. Conclusions

In conclusion, we have successfully developed a highly efficient, transition-metal-free catalytic process for the aerobic oxidation of sulfides with molecular oxygen. It should be noted that the quantitative oxidation of methyl phenyl sulfide to the corresponding sulfoxides with excellent yields can be achieved without acid under mild conditions. A catalyst system is based on molecular bromine as an active catalyst in the presence of very inexpensive NaNO_2_ as co-catalysts. The results showed that reaction with a low concentration of Br_2_ and NaNO_2_ in a small amount of water is highly selective to the desired products without over-oxidation. This reliable non-metal catalytic system for aerobic sulfide oxidation is applicable to a wide range of sulfides, including methyl aryl sulfides and their analogues. 
